# Serine protease *Bm-SP142* was differentially expressed in resistant and susceptible *Bombyx mori* strains, involving in the defence response to viral infection

**DOI:** 10.1371/journal.pone.0175518

**Published:** 2017-04-17

**Authors:** Guohui Li, Qian Zhou, Lipeng Qiu, Qin Yao, Keping Chen, Qi Tang, Zhaoyang Hu

**Affiliations:** Institute of Life Sciences, Jiangsu University, Zhenjiang, China; Wuhan Bioengineering Institute, CHINA

## Abstract

Bm-SP142 is a 35 kDa protease in the silkworm, but its exact functions remain unknown. In this study, sequence alignment revealed that the His-Asp-Ser catalytic triad is embedded in the TAAHC-DIAL-GDSGGP sequence motif, establishing Bm-SP142 as a serine protease. Soluble recombinant GST-BmSP142 was expressed and purified, and serine protease activity was confirmed *in vitro*. RT-qPCR results indicated that *Bm-SP142* was mainly expressed in the middle part of the silkworm midgut, and *Bm-SP142* transcripts were significantly up-regulated at 24 hours post infection (hpi) in *Bm*BDV-resistant strains (798) inoculated with *Bm*BDV and *Bm*NPV-resistant strains (NB) inoculated with *Bm*NPV, but not in *Bm*BDV-susceptible strains (306). Surprisingly, transcripts were significantly down-regulated at 12 hpi in *Bm*NPV-susceptible strains (HuaBa 35) inoculated with *Bm*NPV, compared with healthy silkworms. Recombinant *Bm*NPV treated with purified Bm-SP142 effectively impaired its ability to infect BmN cells, and Bm-SP142 decreases the efficiency of *Bm*NPV and *Bm*BDV propagation in silkworms. Furthermore, overexpression of *Bm-SP142* in BmN cells inhibited viral propagation.

## Introduction

The silkworm is an economically important insect that is raised in developing countries such as China, India and Thailand for the production of silk, and the industry was valued at ~31 billion dollars in 2012 in China alone [1]. The silkworm is also regarded as a model insect of the Order Lepidoptera, due to its convenience for scientific research [2–4]. However, this species is prone to infection by *B*.*mori* densovirus (*Bm*BDV) and *B*.*mori* nucleopolyhedrovirus (*Bm*NPV), resulting in great losses in sericulture [5–7]. Although some strains of silkworm have been developed that are resistant to *Bm*BDV and *Bm*NPV, most strains remain susceptible [8–9].

It is generally accepted that insects lack systemic and specific adaptive immune responses, but they have a highly evolved innate immune system. Like other insects, silkworms also exhibit an effective innate immune response against invading pathogens, which plays an important role in the control and clearance of pathogens [10–11]. The silkworm response to viral infection has attracted extensive attention, and some host proteins such as caspase-1 and V-ATPase are reportedly involved in host resistance to viral infection [12–13].

Serine proteases (SPs) are ubiquitous in all organisms, and silkworms have evolved a particularly abundant and functionally diverse group of proteins that are the predominant digestive enzymes in the insect larval gut [14–15]. Zhao *et al*. [16] reported 51 SP genes in the silkworm genome, many of which may be involved in a variety of physiological processes such as cell signalling, host defences and development. However, it remains unknown whether these SPs are involved in the host defence response against viral infection.

Bao *et al*. [17] first reported that Bm-SP142 is a 35 kDa protease of unknown function in silkworm. In this study, we investigated whether Bm-SP142 was involved in protecting against viral infection. The *Bm-SP142* gene was amplified from the silkworm genome, expressed in *E*.*coli* (BL21/DE3) with an N-terminal GST-tag, and purified in soluble form. The purified Bm-SP142 displayed serine protease activity in *vitro*, and recombinant viruses treated with the protein exhibited impaired infectivity. Furthermore, overexpression of *Bm-SP142* in BmN cells significantly decreased the amount of recombinant virus produced in cells. Additionally, RT-qPCR indicated that Bm-SP142 may be immediately involved in host resistance to viral infection.

## Materials and methods

### Insect, virus,cells and bacterial strains

*Bm*BDV susceptible silkworm strain 306 and-resistant strain 798 (silkworm), and *Bm*NPV susceptible silkworm strain HuaBa 35 and -resistant strain NB are maintained in our laboratory (Institute of Life Science, Jiangsu University, Zhenjiang, China). *Bm*BDV and recombinant *Bm*NPV were propagated in silkworms. BmN cells were cultured at 27°C in TC-100 medium supplemented with 10% Gibco fetal calf serum (Life Technologies). *E*.*coli* strain DH5α was maintained in our laboratory.

### Silkworm rearing and midgut samples preparation

Silkworm larvae (306, 798, HuaBa 35 and NB) were reared on fresh mulberry at 270°C. Each newly-molted 5th-instar larva was inoculated with 5 μl viral stock per os using a pipette. Recombinant *Bm*NPV was directly injected into the hemolymph of silkworms. After 0, 12, 24, 48, 72 and 96 hours postinfection (hpi), silkworm were dissected and midgut samples were collected. Midgut samples from healthy silkworm were used as controls.

### Analysis of *Bm-SP142* transcription

Total RNA was isolated from silkworm midgut tissue using Trizol reagent (Invitrogen), and first-strand cDNA was synthesized with oligo (dT) primers and M-MLV reverse transcriptase (Promega) according to the manufacturer’s instructions. Primer pair Q35-F and Q35-R were used to amplify a 229-bp *Bm-SP142* fragment. The amplified DNA fragment was purified and ligated into the pMD18-T vector to generate recombinant plasmid pMD18-T-Q35. After digestion with *Bam*HI and *Hind*III, the linear fragment was ligated into pFastHTB to produce recombinant plasmid pFastHTB-Q35, which was used as a standard sample.

qPCR was performed in triplicate with 10 μl SYBR Premix Ex TaqTM II (2×), 0.2 μl PCR Forward Primer (20μM), 0.2μl PCR Reverse Primer (20μM), 0.4μl ROX Reference Dye (50×), 2 μl cDNA template and 7.2 μl ddH_2_O as described previously with a modification [18]. Cycling parameters were as follows: 95°C for 30s, followed by 40 cycles of 5 s at 95°C, 31 s at 60°C, 27 s at 72°C. The step of the dissociation curve was followed by 15 s at 95°C, 1min at 60°C, 15 s at 95°C, and 15 s at 60°C. The quantity of PCR product was normalized using the threshold cycle (Ct) value determined by the pFastHTB-Q35 amplification test. Real-time PCR was carried out on an ABI 7300 system (Applied Biosystems,Foster City, CA, USA) using the SYBR PremixEx Taq Kit (Takara) according to the manufacturer’s instructions.

The 5'and 3' ends of the *Bm-SP142* transcript were determined with the 5' Rapid Amplification of cDNA Ends (RLM-RACE) Kit (Ambion) according to the manufacturer's instructions. Briefly, a 45 nt RNA adapter oligonucleotide was ligated to target RNA molecules with leaving a 5'-monophosphate end. The first-strand cDNA was synthesized by reverse transcription with random decamers. The initial PCR was performed with 5'-RACE outer primer and Bm-SP142-R1, and nested PCR was performed to amplify the 5′ end of the *Bm-SP142* transcript with the 5'-RACE inner primer and Bm-SP142-R2. Additionally, 3'-RACE adapter primer ligated with RNA population, which was used to produce the first-strand cDNA by reverse transcription reaction. The first-strand cDNA was used to amplify target DNA with 3'-RACE-F1 and 3'-RACE outer primers, and nested PCR was performed to amplify the 3' end of the *Bm-SP142* transcript with 3'-RACE-F2 and 3'-RACE inner primers. PCR products were purified and cloned into the pMD18-T vector (TaKaRa) for sequencing.

### Expression and purification of recombinant protein

Primer pair 35GST-F and 35GST-R were designed to amplify *Bm-SP142* from a cDNA template of the silkworm genome. After digestion with *Bam*HI and *Xho*I, target DNA was purified and ligated with pGEX-5X-3 to generate recombinant plasmid pGEX-5X-3-*Bm-SP142*. To express Bm-SP142, a freshly transformed colony was selected and cultured in LB medium supplemented with ampicillin (50 μg/ml) at 37°C overnight. A small sample of overnight culture (100 μl) was inoculated into 100 ml fresh LB medium and grown at 37°C with vigorous shaking. When the OD_600_ value reached 0.4, the isopropyl-beta-D-thiogalactopyranoside (IPTG) was added to a final concentration of 0.5 mM to induce the expression of Bm-SP142. All primers used in the study are listed in Table 1.

**Table 1:** Primers used in the study.

Primers	Primer sequence (5'–3')	Restriction site
Q35-F	ACTACAACGACACCGCACAG	---
Q35-R	ATCGGCTTCAGGTCCTCACT	---
35GST-F	ATGGATCCCCATGGCCGGTAAAATGGCGGT	*Bam*HI
35GST-R	ATCTCGAGTCACTCGGCTGCGATGACGTCA	*Xho*I
5'-RACEOuter	GCTGATGGCGATGAATGAACACTG	
5'-RACEInner	CGCGGATCCGAACACTGCGTTTGCTGGCTTTGATG	
Bm-SP142-R1	ATCTCGAGTTAGACCCGGCTGTTCGGGTAGT	
Bm-SP142-R2	CGCTCGAGTTATAGTCTGCAGGGCTGGATGTAACG	
3'-RACE Outer	GCGAGCACAGAATTAATACGACT	
3'-RACE Inner	CGCGGATCCGA4TTA4TACGACTCACTATAGG	
3'-RACE -F1	ATTCTAGGCGGAGTCCAAACCGACGA	
3'-RACE—F2	GTGCAATCTTCACCGTGAGCGGCTA	
Bm-SP142-F	CGGAATTC**ATC**ATGGCCGGTAAA	*Eco*RI
Bm-SP142-R	ATCTCGAGTCACTCGGCTGCGAT	*Xho*I
*ns1 F*	GTTGGTGGTGAAGGGTTTG	
*ns1R*	GGGAGATAGTTTACACTTTGGAG	
GP64-F	TCACTGCTGCCTGATACCC	
GP64-R	ACCATCGTGGAGACGGACTA	
Bm-*actin-F*	TTGCGTCTGGACTTGGC	
Bm-*actin*-R	TTTCGTTTCCGATGGTGA	

Note: underlined letters indicate restriction enzyme digestion sites. Bold letters indicate the KOZAK sequence

After culturing at 20°C for 20 h, cell pellets were harvested by centrifugation (4500×g, 4°C, 10 min) and SDS-PAGE analysis was performed on a 12% gel to estimate the expression level of Bm-SP142. Cell pellets were resuspended in buffer A (140 mM NaCl,2.7mM KCl, 10 mM Na_2_HPO_4_, 1.8 mM KH_2_PO_4_, pH 7.3), the cell suspension was sonicated at 2 W for 1 min on ice, and the supernatant was clarified and by centrifugation. The supernatant was loaded onto a ProteinIso GST Resin affinity column (TRAN), and purification conditions were standardized by optimizing the pH and salt concentration. After thorough washing with buffer A, the fusion protein was eluted with buffer B (50 mM Tris-HCl, 10mM reduced glutathione, pH 8.0). Eluted fractions were subjected to 12% SDS-PAGE analysis and MALDI-TOF-MS analysis.

### Assay of Bm-SP142 serine protease activity *in vitro*

The activity of Bm-SP142 was determined using an ELISA kit for serine proteases (Shanghai Enzyme-linked Biotechnology) according to the manufacturer’s instructions. In brief, 96-well microtiter plates were coated with monoclonal antibodies against insect serine proteases that were provided by the manufacturer. A series of 50 μl volume dilutions of purified Bm-SP142 and 50 μl of standard serine protease sample were added to the plates, and a zero protein and HRP-conjugate reagent were added to blank wells to serve as controls. Plates were mixed by gentle shaking. After incubation for 30 min at 37°C, plates were washed five times with distilled water to remove excess solution and dried by patting with tissue. Next, 50 μl of HRP-conjugate reagent was added to each sample well but not to blank wells. After incubation for 30 min at 37°C, plates were washed five times as described above. Next, 50 μl of chromogen solution A and 50 μl of chromogen solution B were added to each well. After incubation for 10 min, the enzymatic reaction was stopped by adding 50 μl of terminal solution. The optical density (OD) was measured using a microplate reader (Corona Electric, Tokyo, Japan) at a wavelength of 450 nm. The ELISA cutoff value was determined as the average OD_450_ of 30 negative sera plus three standard deviations.

### Analysis of viral propagation in silkworm infected with Bm-SP142-treated virus

Purified Bm-SP142 was incubated with virus, which was subsequently used to infect silkworms, and the level of viral DNA in virus-infected silkworms was determined by qPCR. Briefly, 200 μl of *Bm*BDV or *Bm*NPV was incubated with 200 μl of purified Bm-SP142 for 24 h. Meanwhile, 200 μl of the same viral titre of *Bm*BDV or *Bm*NPV was incubated with 200 μl of blank eluted solution for 24 h as a control. Next, 5 μl of *Bm*BDV was orally administered to 5th instar larvae or subcutaneously injected into 5th instar larvae. Silkworms were infected with the same virus treated with blank eluted solution (control group). A total of 30 silkworms were included in each group.

After cultivation for 48 h with fresh mulberry, silkworms were dissected to collect midgut samples, and total DNA was extracted using a MiniBEST Universal Genomic DNA Extraction Kit Ver.5.0 (TaKaRa). Primer pair GP64-F and GP64-R were used to amplify the *Bm*NPV *GP64* gene from the extracted DNA by qPCR, and primer pair *ns1*F and *ns1*R were used to amplify *ns1*. The amplified DNA fragments were used to indicate the abundance of viral DNA in virus-infected insects.

### Construction of transient expression vector HTB-*Bm-SP142*

HTB-P_ie1_-*Bm-SP142* was constructed using primers Bm-SP142-F and Bm-SP142-R to amplify *Bm-SP142* from the silkworm genome. The target DNA fragment was purified and ligated into the pMD19-T vector and the resulting plasmid was transformed into *E*.*coli* DH5α and propagated in LB medium. *Eco*RI- and *Xho*I-digested pMD19-T-Bm-SP142 was cloned into the pFastHTB-P_ie1_ vector constructed by Li *et al*. [19] to generate the final plasmid HTB-P_ie1_-*Bm-SP142*. The resulting plasmid was verified by sequencing.

### Effect of transient overexpression of Bm-SP142 on viral propagation

To conveniently evaluate the effect of Bm-SP142 on viral propagation, transient overexpression of Bm-SP142 was used to study the propagation of recombinant *Bm*NPV containing GFP in BmN cells. Briefly, 1×10^6^ BmN cells were seeded in six-well plates and incubated at 27°C for 24 h before transfection, 3 μg of HTB-P_ie1_-*Bm-SP142* was mixed with 6 μl Cellfectin Reagent (Invitrogen Life Technology) and used to transfect BmN cells. After 48 h of transfection, recombinant *Bm*NPV containing *gfp* cassette at a multiplicity of infection (MOI) of 5 was used to infect BmN cells. Meanwhile, freshly-seeded BmN cells infected by the same *Bm*NPV titre were used as a blank control, and cells suffering from transfection of HTB-P_ie1_ with no *Bm-SP142* were used as a negative control. After 48 hpi, the amount of GFP present in *Bm*NPV-infected BmN cells was directly counted in the field of vision through fluorescence microscopy. Each transfection was performed in triplicate and the number of GFP were further analyzed statistically.

### Statistical analysis

The abundance valuels of *Bm-SP142* transcript representthe mean ± SD of three assays with 10 larval midguts. A two-way analysis of variance (ANOVA) was used to compare the 306, 798, NB and HuaBa 35 data as well as *Bm*NPV or *Bm*BDV infected larval midguts. The significance between PBS and *Bm*NPV or *Bm*BDV inoculated groups was estimated by student’s t-test.

## Results

### Functional motif analysis of *Bm-SP142* cDNA sequence

**Fig 1:**
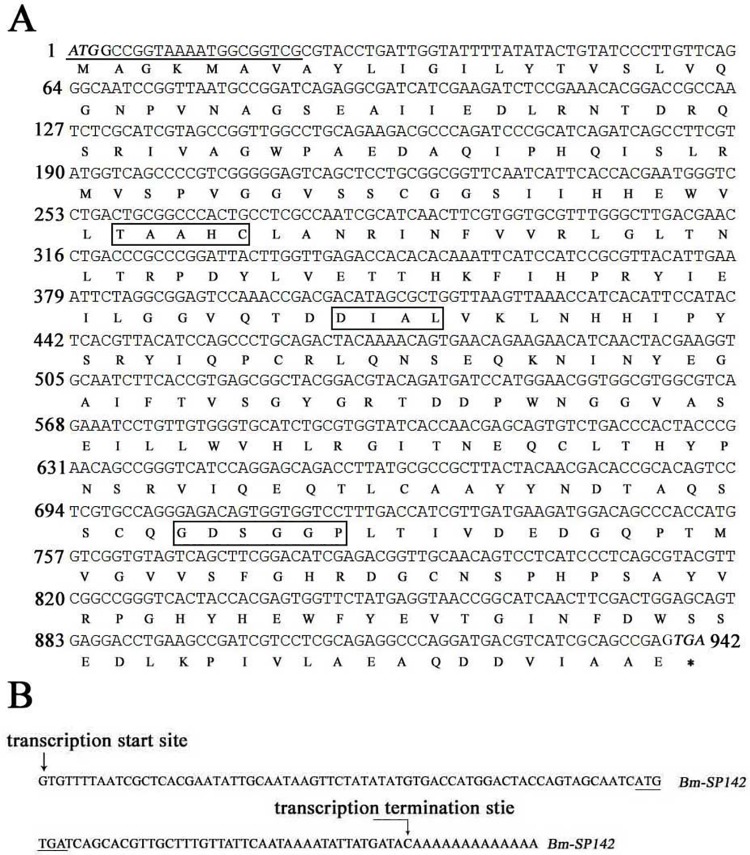
Sequence analysis of *Bm-SP142*. (A) The complete cDNA sequence of *Bm-SP142* and encoded amino acids. The protein sequence is indicated by one letter code below the nucleotide sequence. Three conserved domains are boxed. The start codon (ATG) and stop codon (TGA) are indicated with italic font. The putative signal peptide is underlined with a line. (B) Determination of transcriptional initiation and transcriptional termination sites of *Bm-SP142*. The transcriptional initiation and termination sites of the *Bm-SP142* transcripts are indicated with arrows.

*Bm-SP142* was found to be located at chromosome 16 of silkworm (data not shown). The cDNA sequence of *Bm-SP142* contains an open reading frame (ORF) of 942 bp, which encodes a 313-amino-acid protein with a predicted size of 34.6 kDa and an isoelectric point of 5.35. Three conserved domains of TAAHC, DIAL and GDSGGP was found in the deduced amino acid of Bm-SP142 (Fig 1A), and a typical N-terminal signal peptide with 22 amino acids was predicted in the sequence of Bm-SP142 using SignalP 4.1 Server (http://www.cbs.dtu.dk/services/SignalP/).

To reveal whether *Bm-SP142* contains introns in the genome of silkworm, primer pair 35GST-F and 35GST-R were used to amplify *Bm-SP142* from the silkworm genome. The sequence analysis indicated that no introns were contained in the sequence of *Bm-SP142*. To further reveal the transcription of *Bm-SP142*, 5'-RACE and 3'-RACE was performed to reveal the transcriptional initiation and termination sites in the *Bm-SP142* transcript. The results indicated that the *Bm-SP142* transcript has 68 nt 5’-UTR (untranslated region) and 40 nt 3’-UTR(Fig 1B).

### Alignment of Bm-SP142 and its homologs

**Fig 2:**
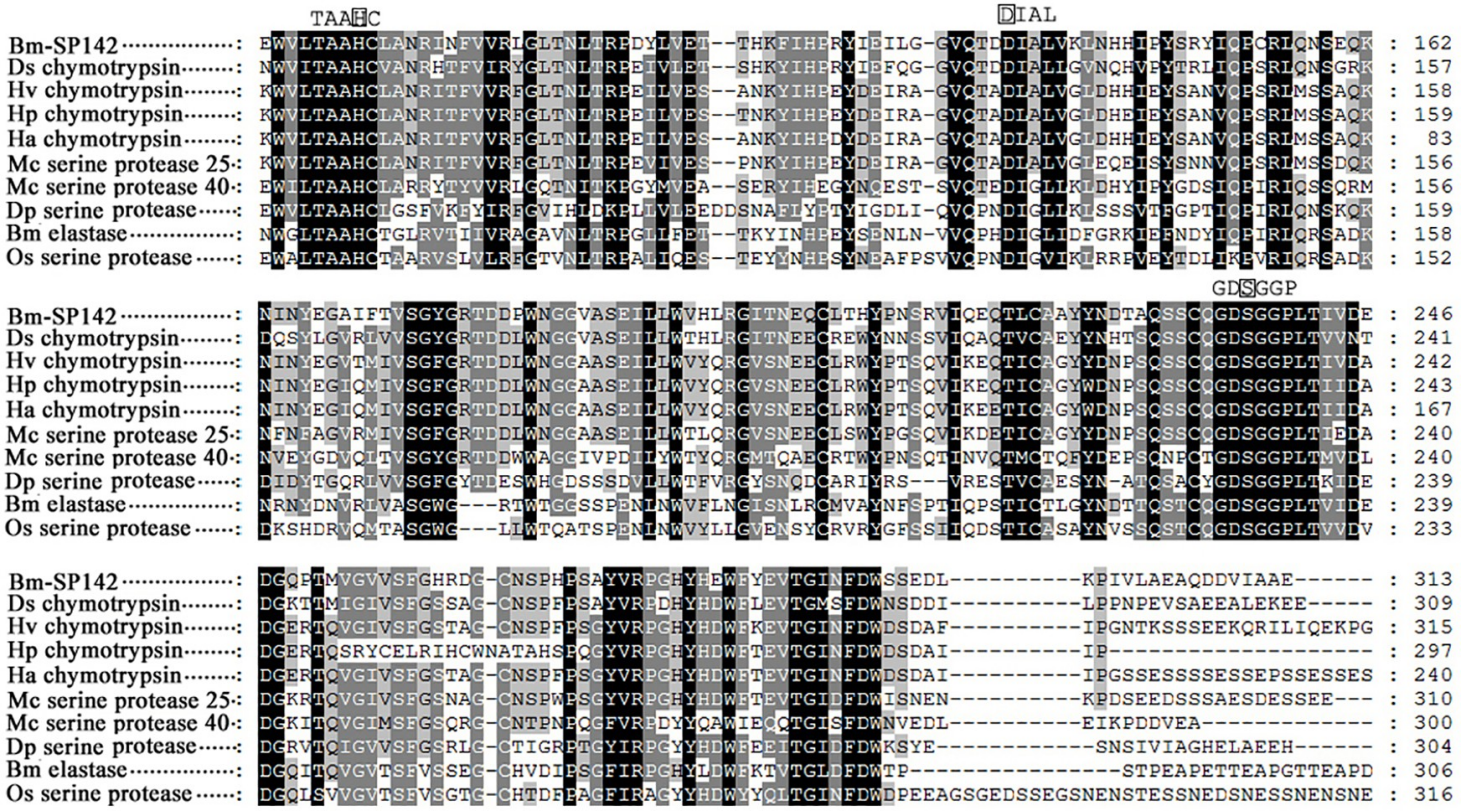
Amino acid sequence alignment of Bm-SP142 and its closest homologs. Identical amino acids are denoted by black shading and similar residues are denoted by grey shading. The active site His (H), Asp (D) and Ser (S) are embedded in the TAAHC-DIAL-GDSGGP sequence. The GenBank number of each sequence was as follows: Bm-SP142 (*Bombyx mori* 35kDa protease precursor, gi|112983142), Ds chymotrypsin (*Diatraea saccharalis* chymotrypsin, gi|411101106), Hv chymotrypsin (*Heliothis virescens*chymotrypsin, gi|390627060), Hp chymotrypsin (*Helicoverpa punctigera* chymotrypsin, gi|54310842), Ha chymotrypsin (*Helicoverpa armigera* chymotrypsin, gi|2463062), Mc serine protease 25 (*Mamestra configurata* serine protease 25, gi|237700800), Mc serine protease 40 (*Mamestra configurata* serine protease 40, gi|304443611), Dp serine protease (*Danaus plexippus* serine protease, gi|357621713), Bm elastase (*Bombyx mori* elastase, gi|512917821), Os serine protease (*Ostrinia nubilalis* serine protease, gi|209395380).

A large number of homologous sequences were obtained from EMBL/GenBank/PIR by a Basic Local Alignment Search Tool (BLAST) search, and multiple sequence alignment was performed using ClustalW and further edited using Genedoc software. The results showed in Fig 2 revealed high sequence similarity between Bm-SP142 and its closest homologs, and three conserved motifs containing the active site His, Asp and Ser residues that form the catalytic triad. Therefore, Bm-SP142 was regarded as a potential serine protease in the silkworm, which may play a possible role in food digestion, embryo development and immune responses.

### Determination of *Bm-SP142* transcriptionin silkworm tissue

**Fig 3:**
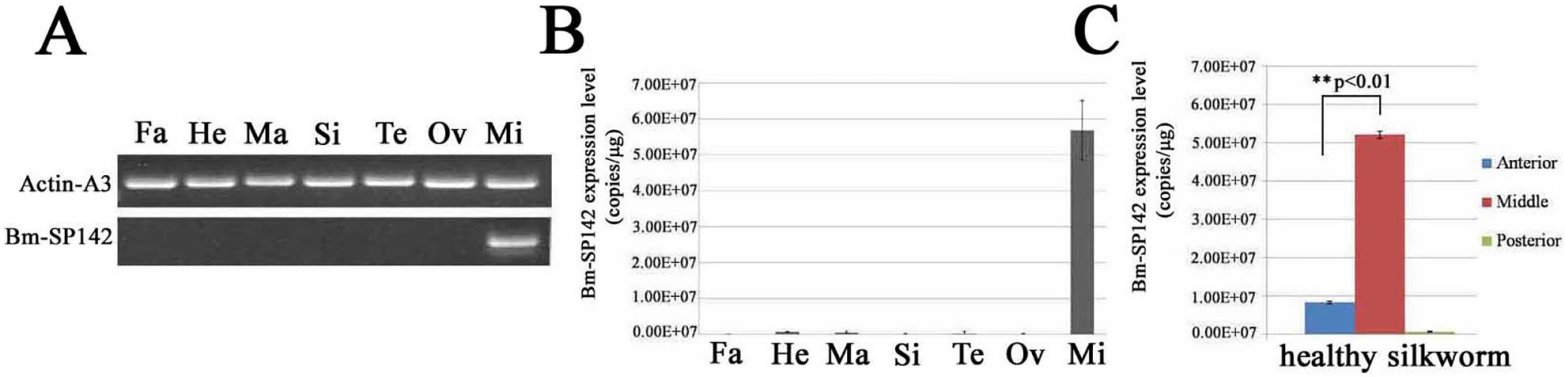
Tissue distribution of *Bm-SP142* in silkworm. (A) RT-PCR analysis of *Bm-SP142* expression in different tissues; (B) Real-Time Quantitative PCR(RT-qPCR) analysis of *Bm-SP142* expression in different tissues; (C) Real-Time Quantitative PCR(RT-qPCR) identification of *Bm-SP142* expression in different parts of the silkworm midgut. Actin-A3 was used as an internal control. Total RNA was extracted from fat body (Fa), hemocytes (He), malpighian tube (Ma), silk gland (Si), testis (Te), ovary (Ov) and midgut (Mi) of individual 5th instar larvae. Triple experiments were performed to calculate the values for relative levels of *Bm-SP142* transcript. Asterisks indicate significant differences compared with control. Error bars indicate standard deviations.

To investigate the expression pattern of *Bm-SP142*, total RNA from 5th instar larvae was extracted from fat body, hemocytes, malpighian tube, silk gland, testis, ovary and midgut, respectively, and subjected to RT-PCR analysis. The result of amplification plots (S1 Fig), dissociation curve of PCR products (S2 Fig) and a standard calibration curve (S3 Fig) were generated, which was used to calculate the copies of *Bm-SP142* transcript in different strains of silkworm. The results in Fig 3A showed that *Bm-SP142* transcription occurred exclusively in the silkworm midgut, and RT-qPCR analysis indicated that 5.8×10^7^ copies of the *Bm-SP142* transcript were present in 1 μg of midgut tissue (Fig 3B). To further examine the distribution of *Bm-SP142* transcription in different parts of the silkworm midgut, additional RT-qPCR analysis was performed, and the results in Fig 3C indicated that the expression level was highest in the middle part of silkworm midgut. The above results indicated that *Bm-SP142* was highly expressed in the midgut of silkworm involved with a potential role in digestion and immune responses.

### *In vitro* catalytic activity of purified Bm-SP142

**Fig 4:**
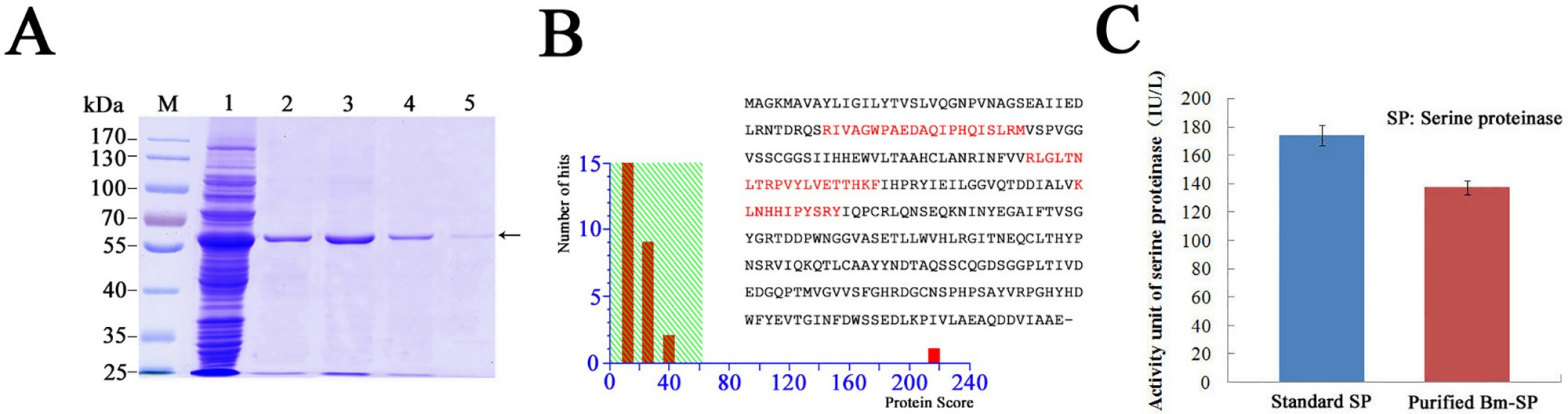
MS identification of recombinant Bm-SP142 and analysis of serine proteinase activity *in vitro*. (A) SDS-PAGE analysis of total protein in *E*.*coli* lysates and purified target protein; lane M, Prestained protein maker; lane 1, Total protein from the lysised *E*.*coli*; lane 2–5, Target GST-fusion protein Bm-SP142 was eluted from ProteinIso^™^ GST Resin affinity column loaded with the lysate of BL21 cells. (B) Analysis of purified Bm-SP142 by MALDI-TOF-MS/MS. Matched peptide sequences are highlighted inred; (C) Serine proteinase activity assay of purified Bm-SP142 *in vitro*. Each bar represents the mean ± SD of three experiments.

Total protein and eluted column fractions from the lysate of *E*.*coli* cells were subjected to SDS-PAGE analysis. The results showed a clear band at ~60 kD in total protein and eluted fractions (Fig 4A), but no similar band in the eluted fractions from control *E*.*coli* cells (data not shown). The ~60 kD protein was further analyzed with MALDI-TOF-MS. The results revealed three peptide fragments with a sufficiently high score that confirmed the protein to be Bm-SP142 (Fig 4B). *In vitro* activity assays confirmed that the ~60 kD protein was a serine proteinase, and as expected, eluted fractions from control *E*.*coli* lysates did not display such activity (data not shown). The activity of recombinant Bm-SP142 was calculated to be 140 IU/L, compared with 180 IU/L for the serine proteinase standard supplied with the assay kit.

### Changes in *Bm-SP142* transcription level following viral infection

**Fig 5:**
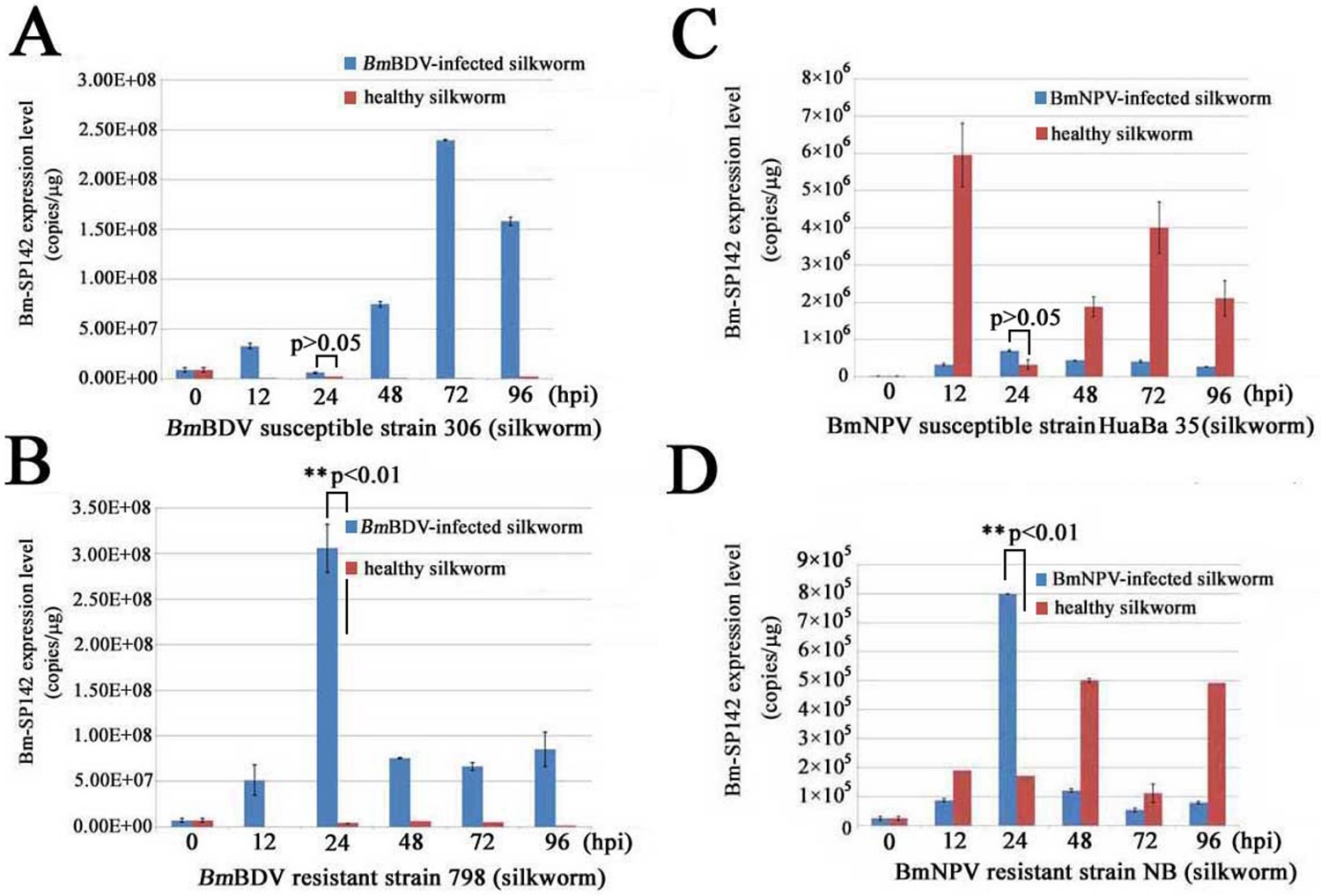
Determination of *Bm-SP142* transcript abundance in different silkworms in response to viral attack. (A) The relative value of *Bm-SP142* transcript abundance in healthy silkworm (strain 306) and *Bm*BDV-infected silkworm (strain 306); (B) The relative value of *Bm-SP142* transcript abundance in healthy silkworm (strain 798) and *Bm*BDV-infected silkworm (strain 798); (C) The relative value of *Bm-SP142* transcript abundance in healthy silkworm (strain HuaBa 35) and *Bm*NPV-infected silkworm (strain HuaBa 35); (D) The relative value of *Bm-SP142* transcript abundance in healthy silkworm (strain NB) and *Bm*NPV-infected silkworm (strain NB). Each bar represents the mean ± SD of three assays.

To investigate the possible role of Bm-SP142 in the responses of silkworm to viral infection, RT-qPCR was carried out to measure *Bm-SP142* transcript abundance in different strains of silkworm. The results indicated that *Bm-SP142* transcripts were expressed at a low level in healthy *Bm*BDV-susceptible strain 306 silkworms and *Bm*BDV-resistant strain 798 silkworms. Meanwhile, total RNA extracted from these strains was used to determine the abundance of *Bm-SP142* transcripts, and RT-qPCR results indicated that the abundance of *Bm-SP142* transcripts was greatly increased in the *Bm*BDV-resistant strain 798 at 24 hpi, but levels declined from 48 hpi to 96 hpi (Fig 5B). By comparison, *Bm-SP142* transcripts were expressed at a low level in *Bm*BDV-susceptible strain 306 at 24 hpi, but increased markedly from 48 hpi to 96 hpi (Fig 5A).

Additionally, the abundance of *Bm-SP142* transcripts was also used to probe the response of silkworm to infection from *Bm*NPV. Total RNA was extracted from susceptible strain HuaBa 35 and *Bm*NPV-resistant strain NB individuals, and the results indicated that *Bm-SP142* transcripts were expressed at a high level in healthy *Bm*NPV-susceptible strain HuaBa 35 silkworms at 12 h (the 5th instar stage), but levels decreased sharply from 24 hpi to 96 hpi in *Bm*NPV-infected HuaBa 35 individuals (Fig 5C). Additionally, *Bm-SP142* transcripts showed a steady increase from 12 h to 96 h at the 5th instar stage in healthy *Bm*NPV-resistant strain NB animals, but were greatly increased in *Bm*NPV-infected NB silkworm at 24 hpi (Fig 5D). There is a significant difference of the *Bm-SP142* abundance (p<0.01) between healthy silkworm (strain 798) and *Bm*BDV-infected silkworm (strain 798) at 24 hpi, and between healthy silkworm (strain NB) and *Bm*NPV-infected silkworm (strain NB) at 24 hpi, respectively. These results indicated that the abundance of *Bm-SP142* transcripts were increased markedly in *Bm*BDV-resistant strain 798 and *Bm*NPV-resistant strain NB silkworms, suggesting Bm-SP142 is likely to play an important role in resistance to viral infection in this species.

### Bm-SP142 decreases the efficiency of viral propagation

**Fig 6:**
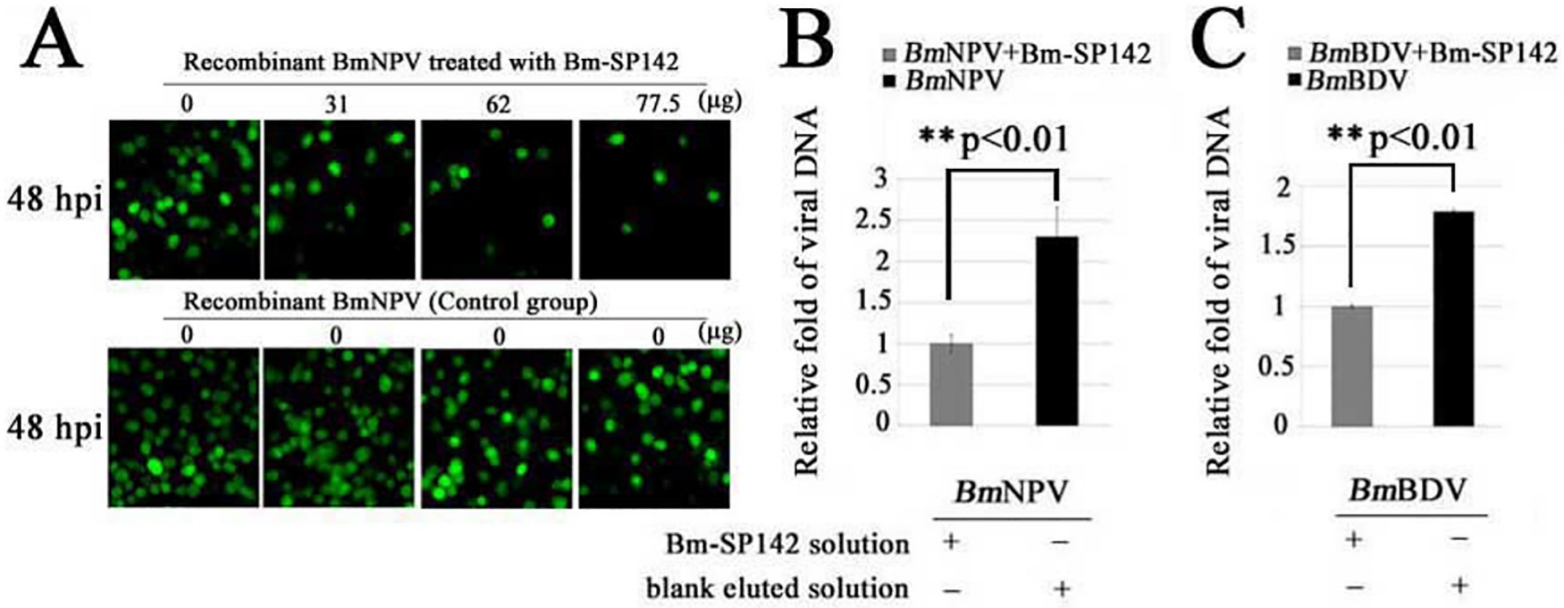
The effect of purified Bm-SP142 on viral propagation. (A) Fluorescence micrographs of BmN cells infected with recombinant *Bm*NPV treated with different amounts of Bm-SP142; (B) Relative number of *Bm*NPV genomes in silkworm infected with Bm-SP142-treated *Bm*NPV compared with *Bm*NPV-treated controls; (C) Relative number of *Bm*BDV genome in silkworm infected with Bm-SP142-treated *Bm*BDV compared with *Bm*NPV-treated controls. Asterisks indicate significant differences compared with control. Each bar represents the mean ± SD of three experiments.

To further examine the inhibitory effect of recombinant Bm-SP142 on viral propagation, equal amounts of recombinant viruses treated with different concentrations of recombinant Bm-SP142 were used to infect BmN cells. The results of fluorescence analysis indicated that the amount of GFP markedly decreased with increasing purified Bm-SP142, while GFP remained unchanged in controls (Fig 6A). Recombinant Bm-SP142 therefore impaired viral propagationin BmN cells.

To evaluate the vitality of *Bm*BDV and *Bm*NPV after treatment with Bm-SP142, the number of copies of each viral genome in silkworms infected with treated viruses was determined. The results indicated that the number of *Bm*NPV genomes in the control group was about 2.3 times higher than that of silkworm infected with Bm-SP142-treated *Bm*NPV (Fig 6B), and 1.8 times higher than that of silkworm infected with Bm-SP142-treated *Bm*BDV (Fig 6C).

### Transient overexpression of *Bm-SP142* inhibits viral propagation

**Fig 7:**
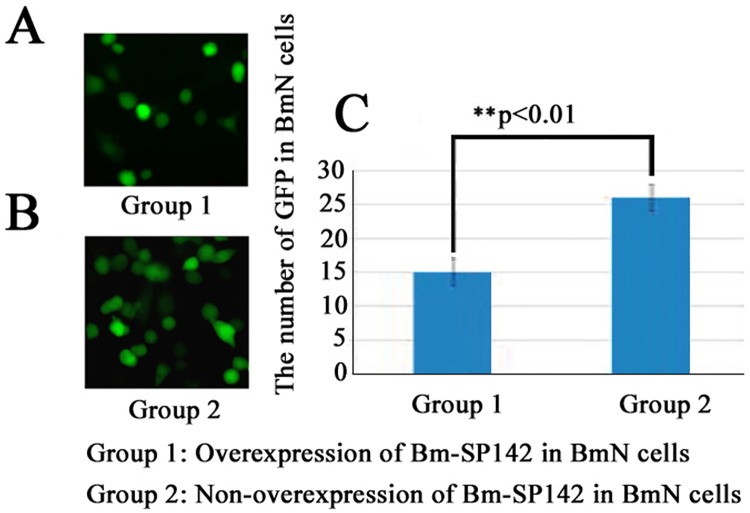
Effect of overexpression of Bm-SP142 on viral propagation. (A) Fluorescence micrograph of BmN cells overexpressing Bm-SP142; (B) Fluorescence micrograph of BmN cells with non-overexpression of Bm-SP142; (C) Statistical analysis of the GFP signal in group 1 and group 2, respectively. Asterisks indicate significant differences compared with control. Each bar represents the mean ± SD of three experiments.

To further elucidate whether Bm-SP142 inhibit viral propagation, overexpression of Bm-SP142 in BmN cells was performed to investigate the effect of Bm-SP142 on the propagation of recombinant *Bm*NPV expressing GFP. The number of GFP signal in BmN cells was counted directly and used to evaluate the efficiency of viral propagation. The results indicated that the GFP signal in Group 1 overexpressing Bm-SP142 was markedly less than that of Group 2 (Fig 7C), and the difference was statistically significant (p<0.01). Bm-SP142 therefore effectively inhibited viral propagation.

## Discussion

Innate immunity plays an important role in sensing pathogens and triggering appropriate biological responses to microbial infection in insects and other invertebrates lacking an acquired immune system [20–21]. In insects, pathogens are recognized as “nonself” and further removed by the synergistic action of both humoral and cellular responses [11, 22], and proteins such as defensin, moricin and lectin were reported to participate in the regulation of immune responses [23–25]. Evidence suggests that serine proteases may be involved in resistance to pathogenic invasion in insects. For example, Qin *et al*. [12] reported that serine proteases may be involved in silkworm defences against attack by *Bm*NPV, and Zou *et al*. [26] reported that serine protease-related genes in the honey bee genome were potentially involved in embryonic development and innate immunity.

Most of silkworm strains tend to be susceptible to viral infection, however, some genetically improved strains in our laboratory are resistant to viral infection. For example, silkworm strain 306 is susceptible to *Bm*BDV infection, and silkworm strain HuaBa 35 is susceptible to *Bm*NPV infection and silkworm strain NB is resistant to *Bm*NPV infection [27–28]. Silkworm strain 798 is resistant to *Bm*BDV infection, which was developed by genetic cross in our laboratory. To date, the molecular mechanism of silkworm resistance to *Bm*NPV or *Bm*BDV infection remains unknown. In the study, serine proteinase Bm-SP142 was confirmed to be differentially expressed in resistant and susceptible *Bombyx mori* strains, and sharply increased in a resistant strain of silkworm suffered from viral infection at 24 hpi (Fig 4). However, whether serine proteases regulate the innate immune responses to viral infection in silkworm remains unclear.

Serine proteases are highly conserved and ubiquitous in eukaryotic and prokaryotic organisms, and they have evolved into an abundant and functionally diverse enzyme group [29–30]. Serine proteases are a major class of digestive proteases, accounting for 95% of digestive activity in Lepidoptera [14]. Zhao *et al*. [16] identified 143 genes in the genome of silkworm as serine proteases, some of which might participate in resistance to pathogenic microorganisms. In this study, recombinant Bm-SP142 was successfully expressed in soluble form in *E*.*coli*, and confirmed to be a novel serine protease. Moreover, changes in *Bm-SP142* transcript abundance in different strains of silkworm was found to be linked to disease resistance; compared with susceptible strains, *Bm-SP142* was more highly expressed in resistant strains at 24 h after viral induction. Expression of *Bm-SP142* was up-regulated significantly within 24 h following pathogenic invasion, strongly indicating a role in activation of the innate immune response. Surprisingly, *Bm-SP142* was also expressed at a relatively high level in healthy silkworm (*Bm*NPV-susceptible strain HuaBa 35), but at a low level in *Bm*NPV-infected silkworm. However, expression of *Bm-SP142* was down-regulated significantly in the susceptible strain after *Bm*NPV induction, suggesting the low expression of *Bm-SP142* was directly involved in the susceptibility of silkworm strain HuaBa 35 to viral infection. Ultimately, it was clear that Bm-SP142 inhibited viral propagation in silkworm.

To further understand the mechanism underlying the inhibitory effects of Bm-SP142, total DNA extracted from silkworm infected with Bm-SP142-treated virus was used to determine the viral genome copy number by qPCR. The results indicated that Bm-SP142 decreased the viability of *Bm*BDV and *Bm*NPV to a remarkable extent. The outer layer of *Bm*BDV and BmNPV virions are comprised of capsid and envelope proteins [31–33], and treatment of viruses with Bm-SP142 presumably resulted in proteolytic cleavages of these capsid and envelope proteins, which decreased their infectivity. We inferred that this proteolytic degradation of virus proteins could impair virus binding to host receptors, and could even completely ablate viral attachment to the silkworm midgut. Consistent with this, the propagation of recombinant *Bm*NPV was clearly inhibited by overexpression of Bm-SP142 in BmN cells (Fig 7). These results suggest Bm-SP142 is an important protein involved in the innate immune response against pathogenic invasion in silkworm.

Previous studies showed that some insect proteins are effective antimicrobial agents. For example, a lipase isolated from the digestive juice of silkworm larvae displays strong antiviral activity against *Bm*NPV, and ODV from *Bm*NPV treated with >2.2 μg of lipase per larva can not propagate in the silkworm host [34]. Similarly, BmAtlastin-n is a member of the dynamin protein superfamily that exhibits antiviral activity against *Bm*NPV in *B*.*mori* [35]. These genes are involved in host innate immunity and are highly expressed in the silkworm midgut. However, unlike Bmlipase-1, expression of *Bm-SP142* gene was upregulated by viral infection in resistant silkworm strains but not in susceptible strains. Transcriptional regulation is involved in some trans-acting factor-mediated regulation of various different responses against pathogens, which may prove useful for identifying these factors in different silkworm strains. However, the antiviral mechanisms of Bm-SP142 in resistant strains of silkworm and the mode of suppressing viral propagation remain unclear. Further research is therefore required.

## Supporting information

S1 Fig The result of amplification plots. (PDF)

S2 Fig The result of PCR products dissociation curve. (PDF)

S3 Fig The result of standard curve. (PDF)

## References

[1] Liu W, Liu E. Analysis on Operation of Chinese Cocoon Silk Industry in 2012 and Prospect in 2013 (I). SILK(Chinese). 2013; 5:72–76.

[2] Goldsmith MR, Shimada T, Abe H. The genetics and genomics of thesilkworm, *Bombyx mori*. Annu Rev Entomol. 2005; 50:71–100. 10.1146/annurev.ento.50.071803.130456 15355234

[3] Duan J, Li R, Cheng D, Fan W, Zha X, Cheng T, et al SilkDB v2.0: a platform for silkworm (*Bombyx mori*) genome biology. Nucleic Acids Res. 2010; 38:453–456. 10.1093/nar/gkp801 PMC2808975 19793867

[4] Nwibo DD, Hamamoto H, Matsumoto Y, Kaito C, Sekimizu K. Current use of silkworm larvae (*Bombyx mori*) as an animal model in pharmaco-medical research.Drug Discov Ther.2015; 9:133–135. 10.5582/ddt.2015.01026 25994065

[5] Hu Z, Zhang X, Liu W, Zhou Q, Zhang Q, Li G, et al Genome segments accumulate with different frequencies in *Bombyx mori* bidensovirus. J Basic Microbiol. 2016; 56(12):1338–1343. 10.1002/jobm.201600120 27160646

[6] Hu Z, Li G, Li G, Yao Q, Chen K. *Bombyx mori* bidensovirus: The type species of the new genusBidensovirus in the new family Bidnaviridae. Chinese Science Bulletin. 2013; 58:4528–4532.

[7] Jiang L, Xia Q. The progress and future of enhancing antiviral capacity by transgenic technology in the silkworm *Bombyx mori*. Insect Biochem Mol Biol. 2014; 48:1–7. 10.1016/j.ibmb.2014.02.003 24561307

[8] Jiang L, Wang G, Cheng T, Yang Q, Jin S, Lu G, et al Resistance to *Bombyx mori* nucleopolyhedrovirus via overexpression of an endogenous antiviral gene in transgenic silkworms. Arch Virol. 2012; 157:1323–1328. 10.1007/s00705-012-1309-8 22527866

[9] Cheng Y, Wang XY, Du C, Gao J, Xu JP. Expression analysis of several antiviral related genes to *Bm*NPV in different resistant strains of silkworm, *Bombyx mori*. J Insect Sci. 2014; 14:76 10.1093/jis/14.1.76 25373223 PMC4212868

[10] Fujiyuki T, Hamamoto H, Ishii K, Urai M, Kataoka K, Takeda T, et al Evaluation of innateimmune stimulating activity of polysaccharides using asilkworm (*Bombyx mori*) muscle contraction assay. DrugDiscov Ther. 2012; 6:88–93. 22622018

[11] Ishii K, Hamamoto H, Kamimura M, Nakamura Y, Noda H, Imamura K, et al Insect cytokine paralytic peptide (PP) induces cellular and humoral immune responses in the silkworm *Bombyx mori*. J Biol Chem. 2010; 285:28635–42. 10.1074/jbc.M110.138446 20622022 PMC2937889

[12] Qin L, Xia H, Shi H, Zhou Y, Chen L, Yao Q, et al Comparative proteomic analysis reveals that caspase-1 and serine protease may be involved in silkworm resistance to *Bombyx mori* nuclear polyhedrosis virus. J Proteomics. 2012; 75:3630–3638. 10.1016/j.jprot.2012.04.015 22546490

[13] Lü P, Xia H, Gao L, Pan Y, Wang Y, Cheng X, et al V-ATPase Is Involved in Silkworm Defense Response against *Bombyx mori* Nucleopolyhedrovirus. PLoS One. 2013; 8:e64962 10.1371/journal.pone.0064962 23823190 PMC3688796

[14] Srinivasan A, Giri AP, Gupta VS. Structural and functional diversities in lepidopteran serine proteases. Cell Mol Biol Lett. 2006; 11:132–154. 10.2478/s11658-006-0012-8 16847755 PMC6275901

[15] Page MJ, Di Cera E. Serine peptidases: classification, structure and function. Cell Mol Life Sci. 2008; 65:1220–36. 10.1007/s00018-008-7565-9 18259688 PMC11131664

[16] Zhao P, Wang GH, Dong ZM, Duan J, Xu PZ, Cheng TC, et al Genome-wide identification and expression analysis of serine proteases and homologs in the silkworm *Bombyx mori*. BMC Genomics. 2010; 11:405 10.1186/1471-2164-11-405 20576138 PMC2996933

[17] Bao YY, Chen LB, Wu WJ, Zhao D, Wang Y, Qin X, et al Direct interactions between bidensovirus *Bm*DNV-Z proteins and midgut proteins from the virus target *Bombyx mori*. FEBS J. 2013; 280:939–949. 10.1111/febs.12088 23216561

[18] Li WX, Yao ZJ, Sun LN, Hu WJ, Cao JJ, Lin WX, et al Proteomics analysis reveals a potential antibiotic cocktail therapy strategy for *Aeromonashydrophila* infection in biofilm. *J* Proteome Res. 2016; 15:1810–1820. 10.1021/acs.jproteome.5b01127 27110028

[19] Li G, Li M, Xu W, Zhou Q, Hu Z, Tang Q, et al Regulation of *Bm*BDV NS1 by phosphorylation: Impact of mutagenesis at consensus phosphorylation sites on ATPase activity and cytopathic effects. J Invertebr Pathol. 2016; 133:66–72. 10.1016/j.jip.2015.12.006 26686834

[20] Viljakainen L. Evolutionary genetics of insect innate immunity.Brief Funct Genomics. 2015; 14:407–412. 10.1093/bfgp/elv002 25750410 PMC4652032

[21] Marques JT, Imler JL. The diversity of insect antiviral immunity: insights from viruses. Curr Opin Microbiol. 2016; 32:71–76. 10.1016/j.mib.2016.05.002 27232381 PMC4983534

[22] Ishii K, Hamamoto H, Sekimizu K. Paralytic peptide: an insect cytokine that mediates innate immunity. Arch Insect Biochem Physiol. 2015; 88:18–30. 10.1002/arch.21215 25521626

[23] Lee YS, Yun EK, Jang WS, Kim I, Lee JH, Park SY, et al Purification, cDNA cloning and expression of an insect defensin from the great wax moth, Galleria mellonella. Insect Mol Biol, 2004; 13:65–72. 14728668 10.1111/j.1365-2583.2004.00462.x

[24] Yamakawa M, Tanaka H. Immune proteins and their gene expression in the silkworm, *Bombyx mori*. Dev Comp Immunol. 1999; 23:281–289. 10426422 10.1016/s0145-305x(99)00011-7

[25] Shi XZ, Kang CJ, Wang SJ, Zhong X, Beerntsen BT, Yu XQ. Functions of Armigeres subalbatus C-type lectins in innate immunity. Insect Biochem Mol Biol. 2014; 52:102–114. 10.1016/j.ibmb.2014.06.010 25014898 PMC4143534

[26] Zou Z, Lopez DL, Kanost MR, Evans JD, Jiang H. Comparative analysis of serine protease-related genes in the honey bee genome: possible involvement in embryonic development and innate immunity. Insect Mol Biol. 2006; 15:603–614. 10.1111/j.1365-2583.2006.00684.x 17069636 PMC1761132

[27] Zhou Y, Gao L, Shi H, Xia H, Gao L, Lian C, et al Microarray analysis of gene expression profile in resistant and susceptible *Bombyx mori* strains reveals resistance-related genes to nucleopolyhedrovirus. Genomics. 2013; 101(4):256–62 10.1016/j.ygeno.2013.02.004 23434630

[28] Chen HQ, Yao Q, Bao F, Chen KP, Liu XY, Li J, et al Comparative proteome analysis of silkworm in its susceptibility and resistance responses to *Bombyx mori* densonucleosis virus. Intervirology. 2012; 55(1):21–8. 10.1159/000322381 21242662

[29] Ruiz-Perez F, Nataro JP. Bacterial serine proteases secreted by the autotransporter pathway: classification, specificity, and role in virulence. Cell Mol Life Sci. 2014; 71:745–770. 10.1007/s00018-013-1355-8 23689588 PMC3871983

[30] Veillard F, Troxler L, Reichhart JM. Drosophila melanogaster clip-domain serine proteases: Structure, function and regulation. Biochimie. 2016; 122:255–269 10.1016/j.biochi.2015.10.007 26453810

[31] Li G, Hu Z, Guo X, Li G, Tang Q, Wang P, et al Identification of *Bombyx mori*bidensovirus VD1-ORF4 reveals a novel protein associated with viral structural component. Curr Microbiol. 2013; 66:527–534. 10.1007/s00284-013-0306-9 23328902

[32] Lv M, Yao Q, Wang Y, Liu X, Liu H, Huang G, et al Identification of structural proteins of *Bombyx mori* parvo-like virus (China Zhenjiang isolate). Intervirology. 2011; 54:37–43. 10.1159/000318888 20689315

[33] Tang Q, Li G, Yao Q, Chen L, Lv P, Lian C, et al Bm91 is an envelope component of ODV but is dispensable for the propagation of *Bombyx mori* nucleopolyhedrovirus.J Invertebr Pathol. 2013; 113:70–77. 10.1016/j.jip.2013.01.006 23391406

[34] Ponnuvel KM, Nakazawa H, Furukawa S, Asaoka A, Ishibashi J, Tanaka H, et al lipase isolated from the silkworm *Bombyx mori* shows antiviral activity against nucleopolyhedrovirus. J Virol. 2003; 77:10725–10729. 10.1128/JVI.77.19.10725-10729.2003 12970462 PMC228431

[35] Liu TH, Dong XL, Pan CX, Du GY, Wu YF, Yang JG, et al A newly discovered member of the Atlastin family, BmAtlastin-n, has an antiviral effect against *Bm*NPV in *Bombyx mori*. Sci Rep. 2016; 6:28946 10.1038/srep28946 27353084 PMC4926086

