# Supplementary figures and images for: Serine protease Bm-SP142 was differentially expressed in resistant and susceptible Bombyx mori strains, involving in the defence response to viral infection
Source: PLoS One. 2017 Apr 17;12(4):e0175518. doi: 10.1371/journal.pone.0175518 (PMC5393580; doi:10.1371/journal.pone.0175518)

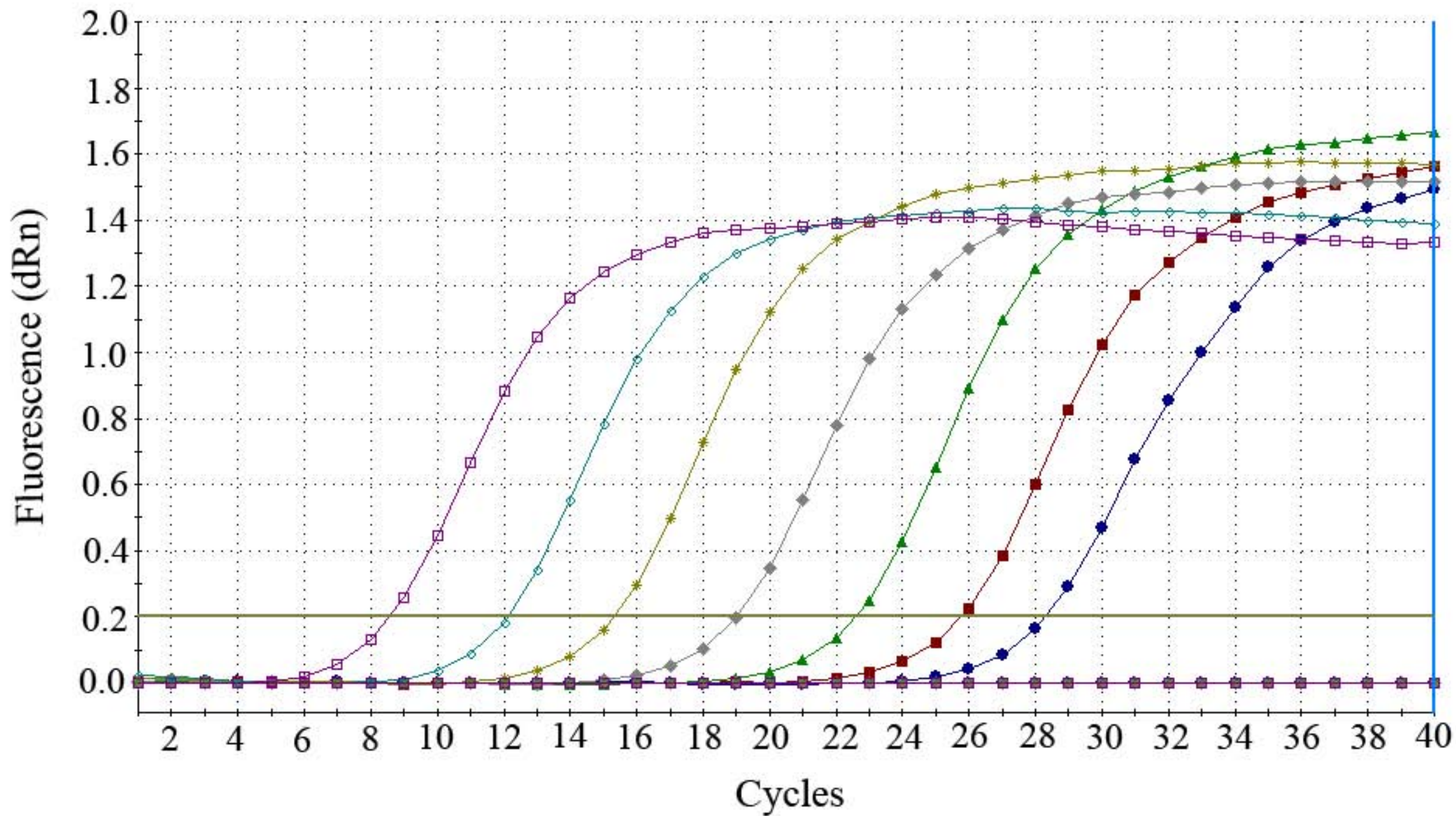

Supplement: S1 Fig — (PDF) [file pone.0175518.s001.pdf]

Fluorescence(-Rn'(T))

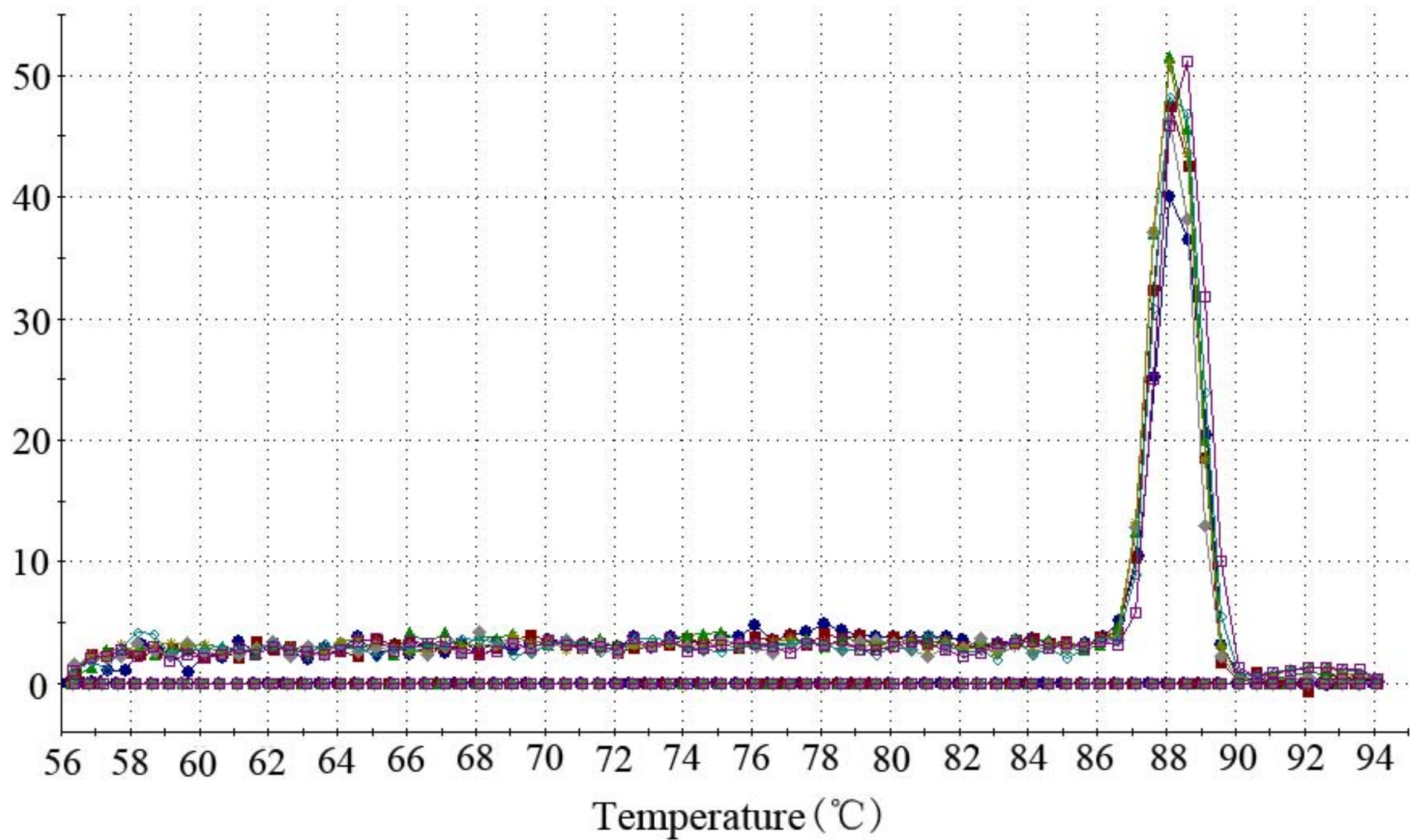

Supplement: S2 Fig — (PDF) [file pone.0175518.s002.pdf]

$$Y = -3.373 \cdot \log(X) + 35.76; R^2: 0.998; \text{Eff.} = 97.9\%$$

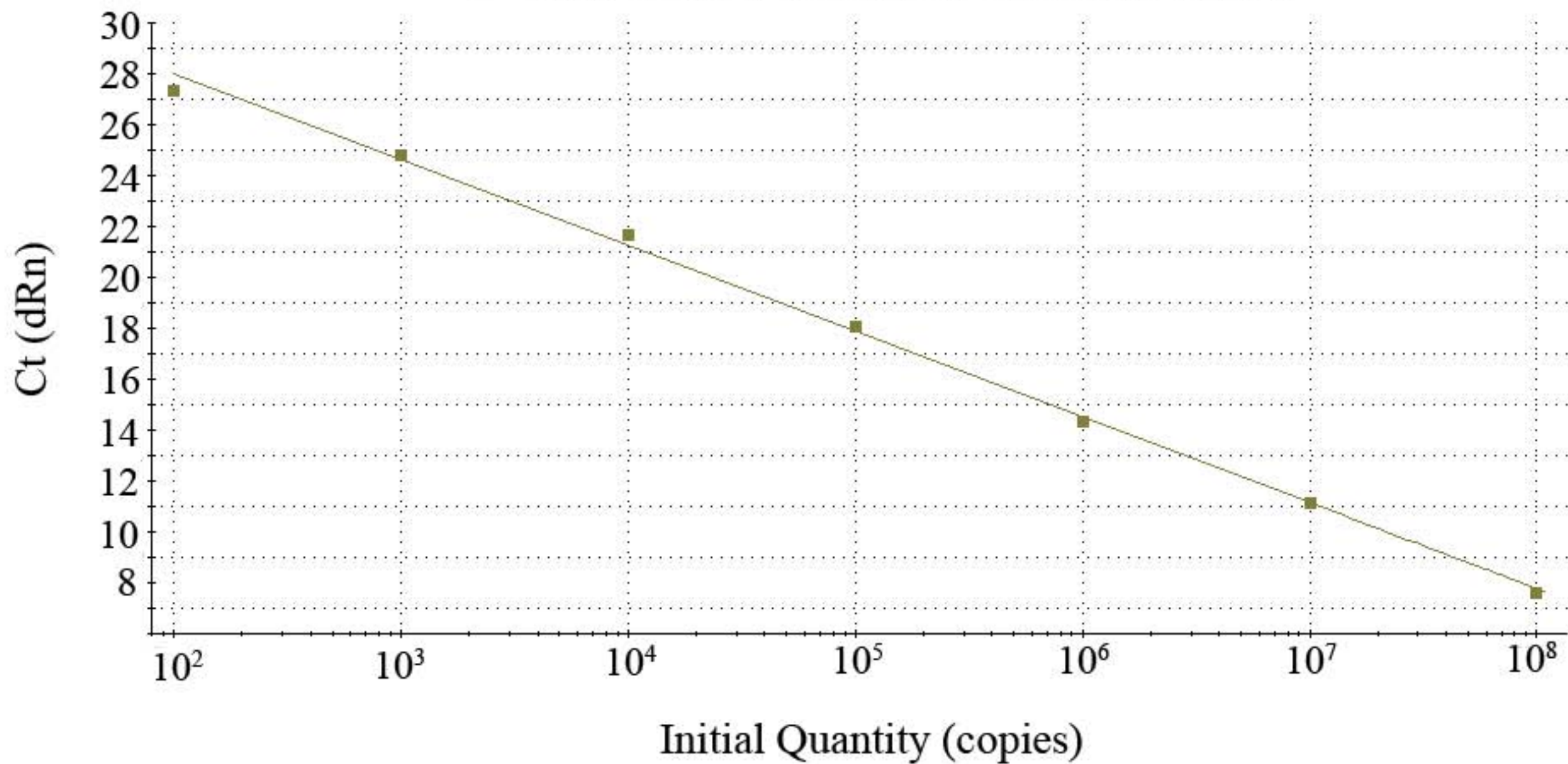

Supplement: S3 Fig — (PDF) [file pone.0175518.s003.pdf]
